# Vessel Co-option: A Promising Therapeutic Strategy in Oral Squamous Cell Carcinoma

**DOI:** 10.7759/cureus.79572

**Published:** 2025-02-24

**Authors:** Shristi Butta

**Affiliations:** 1 Pathology, Institute of Post Graduate Medical Education and Research (IPGMER) and Seth Sukhlal Karnani Memorial (SSKM) Hospital, Kolkata, IND

**Keywords:** aggressive nature, ki67, oral squamous cell carcinoma, targeted therapeutics, vessel co-option

## Abstract

Background

Vessel co-option is the mechanism by which cancer cells take over existing blood vessels for their own growth and metabolism. It is often associated with treatment failure and metastasis in tumors of the brain, lung, liver, and skin. Limited studies have highlighted the role of vessel co-option in oral squamous cell carcinoma (OSCC). This study aims to determine the association between vessel co-option in OSCC and the Ki67 labeling index (LI) to ascertain its prognostic role.

Methodology

In this retrospective study, small biopsy specimens were sent and histopathologically processed to prepare formalin-fixed paraffin-embedded blocks. Only cases diagnosed as OSCC were included in the study. Immunohistochemistry for CD34, p40, and Ki67 was performed.

Results

The study included 39 OSCC patients. The majority (n = 27; 69.23%) of the patients were in the age group of 31-50 years. The male-to-female ratio was 3:1. Tongue (n = 15; 38.46%) was the most common site, followed by the floor of the mouth (n = 11; 28.21%). The majority (n = 25; 64.10%) of the cases were classified as moderately differentiated squamous cell carcinoma. Microscopically, vessel co-option was noted in 71.79% (n = 28) of the cases. Immunohistochemically, Ki67 LI was >10% in 41.03% (n = 16) of the cases. A statistically significant association was found between Ki67 LI and vessel co-option (p = 0.0013).

Conclusions

Vessel co-option is a potential determinant of the aggressive potential of OSCC. Histopathological assessment of vessel co-option in conjunction with the Ki67 proliferative index has promising potential for assessing the prognosis of OSCC.

## Introduction

Oral squamous cell carcinoma (OSCC) is the third leading cause of cancer in India and the sixth most common cancer worldwide [1,2]. It accounts for 90% of all oral cancers [3]. Surgery is the mainstay of treatment for OSCC but has aesthetic concerns. It is often conjugated with postoperative adjuvant therapy [4]. Other treatment modalities include chemoradiotherapy and immunotherapy.

Neo-angiogenesis is the process of sprouting new blood vessels from existing vessels. It is considered the main factor responsible for tumor growth and metabolism. Growth of a tumor beyond 2 mm^2^ requires neo-angiogenesis. Failure of neo-angiogenesis leads to restricted tumor growth and tumor necrosis [5]. It also serves as a target for anti-angiogenic therapies such as bevacizumab. Despite its widespread use, bevacizumab treatment is associated with tumor recurrence and therapeutic resistance. This emphasizes uncovering other underlying angiogenic pathways that result in treatment failure in OSCC.

Vessel co-option is the mechanism by which cancer cells take over existing blood vessels in the vicinity for their own growth and metabolism. Cancer cells migrate between co-opted vessels in the neighboring non-tumorous area to eventually infiltrate into that area and grow [6]. Vessel co-option was first reported in lung cancer. However, any tumor can show vessel co-option [7].

Co-opted vessels differ from neo-angiogenic vessels. Neo-angiogenic vessels are typically irregular, thin-walled, disorganized, and leaky, while co-opted vessels represent existing normal vasculature and are more mature and organized. Histologically, neo-angiogenic vessels are thin-walled with narrow lumen and have incomplete pericyte distribution, while co-opted vessels are relatively thick-walled with larger lumen and have a complete pericytic distribution. Immunohistochemically, neo-angiogenic vessels are weakly positive for smooth muscle actin (SMA), while co-opted vessels are strongly positive for SMA.

Limited studies have illustrated the role of vessel co-option in OSCC. Few studies have developed infra-red theranostic agents for targeting tumor-associated vessels in OSCC [8]. However, to our knowledge, no study to date has ascertained the role of vessel co-option in proliferating OSCC cells by histopathological examination. This study is distinctive and one of its kind. The prime objective of this study was to determine the association between vessel co-option (as ascertained by a histopathologic evaluation) and the proliferative potential of OSCC (as ascertained by the Ki67 labeling index (LI)).

## Materials and methods

This retrospective study was conducted in the Department of Pathology of a tertiary care hospital. Small biopsy specimens taken from lesions involving the oral mucosa, tongue, floor of the mouth, and oropharynx were sent to the Department of Pathology. The samples were histopathologically processed. Formalin-fixed paraffin-embedded (FFPE) blocks were prepared. Blocks prepared over the last 15 months were retrieved from the archives. Clinical and imaging data was obtained from the relevant data records. Routine hematoxylin and eosin staining was done on the retrieved FFPE blocks. Slides were microscopically analyzed and only cases diagnosed as OSCC were included in the study.

Subsequently, immunohistochemistry for CD34, p40, and Ki67 was performed on the FFPE blocks. CD34 was used to highlight the endothelial cells of the vessels, p40 was used to confirm the squamous nature of the tumor cells, and Ki67 was used as a marker of the proliferation of tumor cells. Rabbit monoclonal CD34 antibody, rabbit monoclonal p40 antibody, and rabbit monoclonal Ki67 antibody were used as the primary antibodies.

For immunohistochemistry, 5 µm thick sections were cut from the FFPE blocks and deparaffinized. Endogenous peroxidase activity was blocked using hydrogen peroxide incubation for 20 minutes. Further, the sections were treated with tris-buffered saline to prevent non-specific binding. Overnight incubation with the primary antibody (monoclonal CD34, p40, Ki 67) was done to allow the binding of the primary antibody to its sites. The sections were further incubated with enzyme-linked secondary antibodies for one hour. Subsequently, deoxyaminobenzidine chromogen was added and allowed to stand for 10 minutes. Sections were then treated with water and counterstained with hematoxylin stain. Then, sections were dehydrated in graded alcohol, cleared in xylene, and mounted with dibutylphthalate polystyrene xylene. CD34 highlighted the endothelial cells, and p40 confirmed the squamous nature of the tumor cells. Ki67 LI was calculated as a percentage of positive tumor cells among 1,000 tumor cells. A Ki67 LI <5% was considered low, 5-9% was considered intermediate, and >10% was considered high. The tonsil was used as a positive control for CD34 and Ki67 antibodies, while known cases of lung squamous cell carcinoma were used as a positive control for the p40 antibody.

Statistical analysis was conducted using the software Epi Info version 7.2.6.0. A p-value <0.05 was considered to be statistically significant.

## Results

The study included 39 cases of OSCC. Overall, 69.23% (n = 27) of the patients were in the age group of 31-50 years, while 17.95% (n = 7) of the patients were in the age group of 51-70 years. Further, 10.26% (n = 4) were aged <30 years, while only 2.56% (n = 1) were aged >70 years. There was a male predilection with a male-to-female ratio of 3:1.

**Figure 1:**
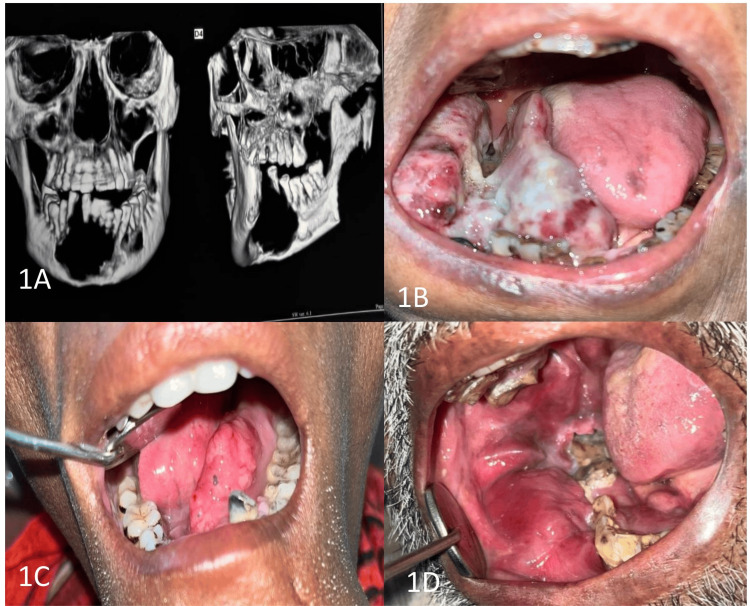
(A) Cone-beam computed tomography scan showing the destruction of the underlying bone in a case of oral squamous cell carcinoma of the floor of the mouth. (B, C) Clinical picture of the tumor involving the floor of the mouth. (D) Clinical picture of the tumor involving the right buccal mucosa.

A history of tobacco consumption was noted in 64.10% (n = 25) of the cases. The majority of the cases involved the tongue (38.46%; n = 15), followed by the floor of the mouth (28.21%; n = 11). The rest of the cases involved the oropharynx (20.51%; n = 8) and oral mucosa (12.82%; n = 5) (Figure 1).

**Figure 2:**
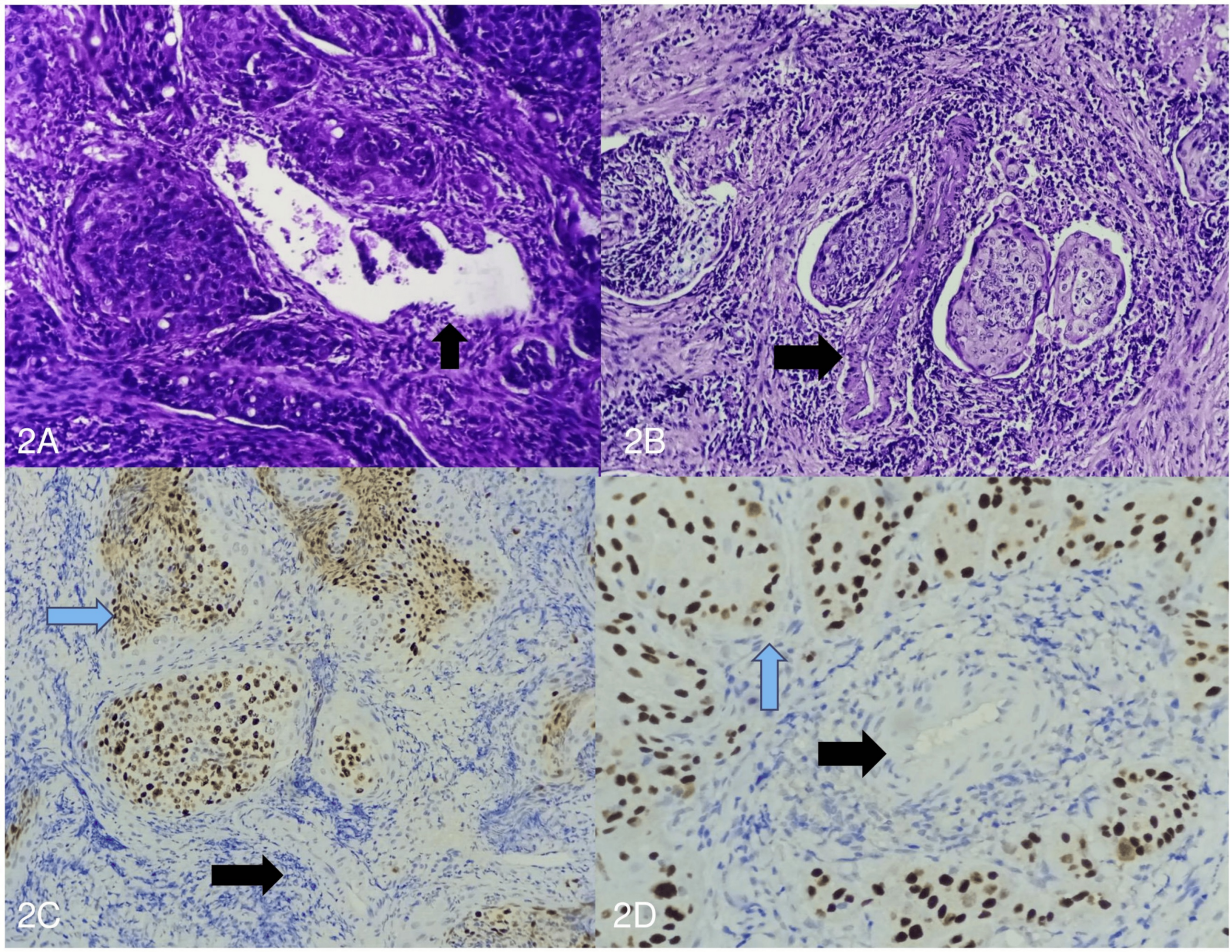
(A, B) Photomicrograph showing the squamous nests of moderately differentiated squamous cell carcinoma around the co-opted vessel (highlighted by a black arrow) (hematoxylin and eosin, 200×). (C,D) Photomicrograph showing squamous nests (highlighted by a blue arrow) around the co-opted vessel (highlighted by a black arrow) (p40, 200×).

Microscopically, 64.10% (n = 25) of the cases were classified as moderately differentiated squamous cell carcinoma, while 28.21% (n = 11) were well-differentiated squamous cell carcinoma. Only 7.69% (n = 3) of the cases were classified as poorly differentiated squamous cell carcinoma. Further, microscopically, vessel co-option was noted in 71.79% (n = 28) of the cases, while 28.21% (n = 11) had no co-opted vessels (Figure 2).

**Figure 3:**
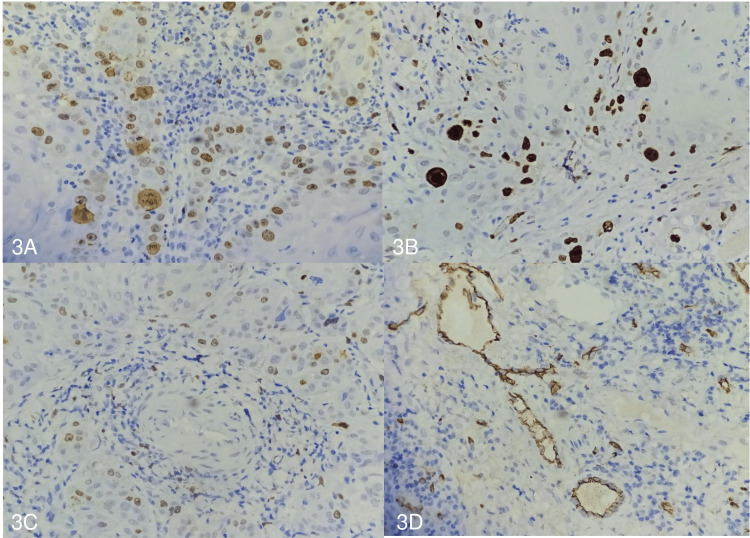
(A) Ki67 labeling index >10% (Ki67, 200×). (B) Ki67 labeling index of 6-9% (Ki67, 200×). (C) Ki67 labelling index <5% (Ki67, 100×). (D) CD34 highlighting the endothelial cells of the blood vessels (CD34, 200×).

Immunohistochemically, Ki67 LI was >10% in 41.03% (n = 16) of the cases, 6-9% in 30.77% (n = 12) of the cases, and <5% in 28.21% (n = 11) of the cases. A statistically significant association was found between Ki67 LI and vessel co-option (p = 0.0013) (Figure 3, Table 1).

**Table 1:** Sssociation between vessel co-option and Ki67 labeling index. p = 0.0013.

	Ki67 labeling index			
Vessel co-option	<5%	>10%	6–9%	Total
Absent	7	0	4	11
Row%	63.64%	0.00%	36.36%	100.00%
Col%	63.64%	0.00%	33.33%	28.21%
Present	4	16	8	28
Row%	14.29%	57.14%	28.57%	100.00%
Col%	36.36%	100.00%	66.67%	71.79%
Total	11	16	12	39
Row%	28.21%	41.03%	30.77%	100.00%
Col%	100.00%	100.00%	100.00%	100.00%

## Discussion

Angiogenesis plays a significant role in tumor growth and metastasis in all tumors including OSCC. Despite sprouting angiogenesis, growing tumor cells are always nutrient-hungry. This possibly leads to a non-angiogenic mechanism of vascular tropism, also referred to as vessel co-option. Other non-angiogenic mechanisms of tumor growth include vasculogenic mimicry and intussusception [9]. Vessel co-option is a process whereby growing tumor cells use the existing vascular supply to mitigate their hunger. This non-angiogenic mechanism of tumor growth and metastasis has been established in the lung, brain, liver, and lymph nodes [9]. On the other hand, vasculogenic mimicry is due to the inherent plasticity of tumor cells where they form vessel-like structures [10]. Intussusception is a process wherein the existing vessels divide to form daughter vessels to supply for the nutrient-deficient tumor [11]. Of all the three, vessel co-option is the most common.

The major risk factors associated with OSCC include tobacco chewing and alcoholism. The combination of alcohol and tobacco has a synergistic role and increases the risk of head and neck squamous cell carcinoma. Further, human papillomavirus (HPV) and Epstein-Barr virus (EBV) are also notable risk factors. Of these two viruses, HPV is frequently associated with oropharyngeal OSCC [12,13]. Herein, we found tobacco consumption to be associated with a majority of the cases while a history of alcohol intake was not significant. However, in a large cross-sectional study by Eloranta et al. involving 519 patients, it was found that more than half of the patients with OSCC reported either a history of smoking or alcohol intake, or both [14]. Further, regarding the gender predisposition, males were more frequently affected compared to females. These results are consistent with Moreno et al. who reported that the incidence of OSCC was twice as high in males compared to females [15]. Regarding the age of predisposition, the majority of the patients belonged to the age group of 31-50 years, followed by 51-70 years. This is consistent with global data suggesting that there has been a significant rise in the incidence of OSCC in young adults under 45 years of age [16]. This could be attributed to the increased nicotine abuse, especially in young adults, in the form of cigarette or bidi smoking and tobacco or betel quid chewing [17].

Further, regarding the site of involvement, the tongue was the most commonly affected site, followed by the floor of the mouth. However, Al-Rawi et al. in a large study involving 231 OSCC cases found the tongue to be the most common site, followed by the oral mucosa of the cheek. Additionally, they also found that the most common site among smokers was the floor of the mouth [18]. Site-specific molecular pathogenesis has been investigated by several studies in the past. OSCC involving the buccal mucosa and tongue shows variably different levels of downregulation of *p16* and *p21* genes [19]. Additionally, the activity of the telomerase enzyme significantly varies in tumors originating from the floor of the mouth and tongue. Further, the X-linked inhibitor of apoptosis protein expression significantly varies in OSCC originating from the floor of the mouth and tongue, with the expression being lower for tongue squamous cell carcinoma [20,21]. Regarding the histopathological grading, moderately differentiated OSCC was the most common, followed by well-differentiated OSCC. The results were similar to those reported by Yasin et al. [22]. However, Padma et al. reported well-differentiated OSCC to be more common [23].

The treatment of OSCC mainly includes surgery. However, it poses cosmetic concerns and is often denied by the patients. Other treatment modalities include chemoradiotherapy and targeted therapy. Targeted therapy mainly includes immunotherapy (e.g., pembrolizumab) and anti-angiogenic therapy (e.g., bevacizumab). The five-year survival rate of early-stage OSCC is about 80% whereas that of advanced-stage OSCC has been documented to be less than 30% [23]. This is regardless of the advances in the treatment modalities. The reason for this treatment failure could be particularly attributed to the therapeutic resistance and increased tendency to metastasize. Further, vessel co-option could potentially impact the outcome of chemotherapy, radiotherapy, immunotherapy, and surgery. This aspect has been described by Wang et al. in brain tumors wherein vessel co-option has been proposed to be responsible for residual tumor and subsequent tumor recurrence [24]. Herein, vessel co-option was associated with a majority of our cases. On immunohistochemical analysis, the proliferative index was comparatively high in most of the vessel co-opted cases. A statistically significant association was noted between vessel co-option and the proliferative potential of the tumor. Further, vessel co-option could be a potential determinant in stratifying OSCC cases into bevacizumab-responsive and non-responsive groups. Hence, inhibiting vessel co-option in addition to the conventional anti-angiogenic therapy can potentially predict patient prognosis.

Unlike neo-angiogenesis which has framed strategies for assessment, vessel co-option has no specific guidelines; hence, standardization of methodologies is crucial for the better reliability of results [25]. Zhang et al. suggested that vessel co-option could lead to tumor latency [26]. This could be a potential reason for the late diagnosis of OSCC. Further, this observation also suggests that vessel co-option could be a potential strategy used by tumor cells to spread and escape anti-angiogenic therapies in OSCC.

Potential therapeutic strategies targeting vessel co-option are underway. These mainly include the anti-invasive therapies interfering with tumor cell migration along the vessels, tumor-endothelial interaction, and adhesion. Additionally, combining anti-invasive therapies with conventional anti-angiogenic therapy such as bevacizumab can potentially prevent tumor resistance and metastasis [27].

As this study was conducted in a single institution or hospital, the cases included may not be representative of the entire population. In addition, sample size was a constraint. Hence, the observations require further studies to affirm the potential role of vessel co-option in the prognosis of OSCC. Further, the lack of standardization of strategies employed in ascertaining vessel co-option by histopathological examination also needs investigation. Furthermore, there are certain confounders that need to be mentioned. The tumor microenvironment could be a stimulator of vessel co-option in addition to hypoxia, both of which have a potential role in impacting tumor prognosis and treatment resistance.

## Conclusions

Vessel co-option is a potential determinant of the aggressive potential in OSCC. Standardization of strategies to determine vessel co-option could help stratify patients into high-risk and low-risk cases. Histopathological assessment of vessel co-option in conjunction with the Ki67 proliferative index has promising potential for assessing the prognosis of OSCC. Co-opted vessels provide adequate nutrients to the growing tumor and can be potential targets to improve patient prognosis in OSCC. Inhibiting co-opted vessels in addition to the conventional targeted vessels can potentially prevent tumor latency and subsequent tumor metastasis, in turn, improving patient prognosis.
